# Verwendung von forensischer Zahnmedizin zur Identitätsfeststellung. Eine Befragung auf Ebene der Länderpolizei

**DOI:** 10.1007/s00103-023-03769-2

**Published:** 2023-09-27

**Authors:** Monika Bjelopavlovic, Franziska Badt, Karl Martin Lehmann, Katja Petrowski

**Affiliations:** 1 Poliklinik für Zahnärztliche Prothetik und Werkstoffkunde, Unimedizin der Johannes Gutenberg-Universität Mainz, Mainz, Deutschland; 2 Medizinische Psychologie und Soziologie, Johannes Gutenberg-Universität Mainz, Mainz, Deutschland

**Keywords:** Forensische Zahnmedizin, Kriminalpolizei, Primäres Identifizierungsmerkmal, Digitalisierung, Identifizierung, Forensic dentistry, Criminal investigation, Primary identifier, Digitization, Identification

## Abstract

**Hintergrund:**

In Deutschland liegt die Identifizierung unbekannter Toter in der Zuständigkeit der Polizei. Gemäß INTERPOL-Standards werden primäre (DNA, Fingerabdrücke, Zähne) und sekundäre Merkmale (z. B. Tätowierungen) untersucht. Die forensische Zahnmedizin wird als effiziente Methode bereits international eingesetzt. In dieser Studie soll auf Länderebene in Deutschland die Vorgehensweise der Polizei analysiert werden. Untersucht werden angewandte Methoden bei der Identifizierung, die Rolle der forensischen Zahnmedizin, die Zusammenarbeit mit Zahnärzt*innen und mögliche Optimierungsansätze.

**Methoden:**

Mittels eines digitalen Fragebogens sollten in allen Bundesländern für Funde unbekannter Toter zuständige Polizeibeamt*innen zu Identifizierungsmethoden und speziell zur Anwendung forensischer Zahnmedizin befragt werden.

**Ergebnisse:**

85 Sachbearbeiter*innen aus mind. 11 Bundesländern nahmen an der Befragung teil. Die Vorgehensweise stellte sich als dienststellenspezifisch heraus. In 72,6 % der Fälle werden bei der Identifizierung verschiedene Merkmale kombiniert, am häufigsten DNA mit Zahnstatus (37,1 %). Die DNA-Analyse wird am häufigsten verwendet. 62,9 % der Befragten stimmten zu, dass die zahnärztliche Identifizierung „oft“ angewendet wird. Der Anteil der Identifizierungen mittels Zahnstatus wird auf 1,6–8,1 % geschätzt. Für die forensische Zahnmedizin haben 19,4 % eine feste Ansprechperson. Eine digitale Plattform, um Zahnmediziner*innen zu kontaktieren, schätzten 56,5 % als hilfreich ein.

**Diskussion:**

Die forensische Zahnmedizin steht aktuell noch hinter der DNA-Analyse zurück, was sich durch die zunehmende Digitalisierung ändern könnte, wenn z. B. Ante-mortem-Daten zuverlässiger zur Verfügung stehen und Plattformen für den interdisziplinären Austausch geschaffen werden.

**Zusatzmaterial online:**

Zusätzliche Informationen sind in der Online-Version dieses Artikels (10.1007/s00103-023-03769-2) enthalten.

## Abstract (en)

**Background:**

In Germany, the identification of unknown dead persons is the responsibility of the police. According to INTERPOL standards, primary (e.g., DNA, fingerprints, and teeth) and secondary (e.g., tattoos) characteristics are examined. Forensic dentistry is already used internationally as an efficient method. In this study, the approach of state police in Germany was analyzed. The methods used for identification, the role of forensic dentistry, the cooperation with dentists, and possible optimization approaches are investigated.

**Methods:**

By means of a digital questionnaire, police officers competent in all federal states for the discovery of unknown dead bodies were asked about identification methods and specifically about the use of forensic dentistry.

**Results:**

Eighty-five officers from at least 11 federal states participated in the survey. The procedure turned out to be department specific. In 72.6% of the cases, different characteristics are combined in the identification process, most frequently DNA with dental status (37.1%). DNA analysis is used most frequently. Of the respondents, 62.9% agreed that dental identification is used “often.” The percentage of identifications using dental status is estimated to be 1.6–8.1%. For forensic dentistry, 19.4% have a fixed point of contact. A digital platform to contact dentists was estimated to be helpful by 56.5%.

**Discussion:**

Forensic dentistry is currently still lagging behind DNA analysis, which could change through increasing digitalization if, for example, ante-mortem data are more reliably available and platforms for interdisciplinary exchange are created.

## Einleitung

Für die Identifizierung von unbekannten Toten gibt es weltweite INTERPOL-Standards, bei denen die Untersuchung von primären und sekundären Identifizierungsmerkmalen unterschieden wird. Zu den primären Merkmalen gehören DNA, Fingerabdrücke und Zähne [1, 2], zu den sekundären zählen beispielsweise Tätowierungen, mitgeführte Gegenstände und die Bekleidung. Eine visuelle Identifizierung der Opfer, zum Beispiel durch Verwandte, ist nach INTERPOL-Standard (Internationale kriminalpolizeiliche Organisation) unbedingt zu vermeiden, da sie erfahrungsgemäß große Fehlerquellen birgt [3].

Die herausragende Rolle der forensischen Zahnmedizin (forensische Odontologie) konnte in Großschadenslagen wie dem Tsunami in Thailand 2004 eindrücklich aufgezeigt werden, da ein Großteil der Opfer (79 %) allein anhand des Zahnstatus identifiziert werden konnte [4, 5]. Ebenfalls konnte die Mehrzahl der Opfer eines Flugzeugabsturzes bei Zell am See im Jahr 2007 anhand des Zahnstatus eindeutig identifiziert werden [6]. Aus diesen Erfahrungswerten entstand eine internationale Formsprache, die im Schadensfall zu einem länderübergreifenden Verständnis durch gemeinsame Standards und Codierungen führte. Weiterhin kam ein globales Engagement für Identifizierungsverfahren von Katastrophenopfern zustande (DVI: Disaster Victim Identification), um die Prozessketten sicher und effizient zu gestalten [7–9].

In diesem Kontext ist die forensische Zahnmedizin mit sehr charakteristischen und individuellen Befunden in der Mundhöhle auch bei großen Noxen wie Feuer und Wasser sowie bei fortgeschrittenem Verwesungsprozess von hohem Stellenwert [10]. Trotz steigender Mundgesundheit in der deutschen Bevölkerung [11] kann der Zahnstatus aufschlussreich sein, da zur Identifizierung nur wenige Zahnbefunde benötigt werden, was eine groß angelegte Studie aus dem spanischen Raum mit 3166 Individuen zeigen konnte: Bei nur 4 Merkmalen (fehlend, restauriert, unrestauriert, Krone) und nur 16 vorhandenen Zähnen (vollbezahntes menschliches Gebiss: 32 Zähne) besteht eine sehr geringe Wahrscheinlichkeit (0,05 %), dass 2 Individuen eine identische Kombination aufweisen [12].

Im klinischen Alltag arbeitet man bei der Befundung mit weitaus mehr als 4 Begriffen. Einbezogen werden Flächenangaben, Materialien (wie zum Beispiel von Füllungen, Kronen oder Prothesen), Zahnstellungen und Besonderheiten wie das Vorliegen von Abriebspuren bei Bruxismus („Zähneknirschen“). Diese hochindividuellen Merkmale können zur Identitätsaufklärung beitragen [13–15]. Des Weiteren können Röntgenbilder einbezogen werden. Sie liefern zusätzliche und oft sehr prägnante Informationen zur Post-mortem-Identifizierung [16–18]. Die zahnärztliche Radiologie wird im klinischen Alltag zur Diagnostik, zur postoperativen Kontrolle und zur Nachsorge beim Eingliedern von implantologischen Suprakonstruktionen (Zahnersatz auf Implantaten) und bei Verlaufskontrollen eingesetzt [19–21]. Nach den Terroranschlägen 2015 in Paris wurde das standardisierte Vorgehen in Frankreich bei der Identifizierung von Toten angepasst. Es werden nun Ganzkörper-CTs der Leichen gefordert und die Wichtigkeit von dentalem Röntgen bei Großschadenslagen hervorgehoben [22, 23].

Neben den sehr bekannten primären Identifizierungsmerkmalen DNA und Fingerabdruck liegt die Hinzunahme oder alleinige Identifizierung über den Zahnstatus in Deutschland wie nachfolgend beschrieben im Zuständigkeitsbereich der ermittelnden Polizeibeamten*innen, die sich mit der Identitätsfeststellung auseinandersetzen. Anhand kriminalpolizeilicher Erfahrungswerte entscheiden sie, welche Methoden in Auftrag gegeben werden: Fingerabdruck und/oder DNA und/oder Zahnstatus. Die Entscheidung kann durch verschiedene Faktoren beeinflusst werden, zum Beispiel das Vorliegen eines fortgeschrittenen Verwesungszustands oder das Fehlen von Körperteilen.

Die Akquise antemortaler Daten, die zum Vergleich mit postmortal gewonnenen Daten vorliegen müssen, wird meist im Rahmen einer Vermisstenanzeige im Vorfeld in Gang gesetzt. Dabei werden der Zahnstatus, Röntgenbilder oder Zahnmodelle von der/dem letztbekannten behandelnden Zahnärzt*in angefordert, wie auch Vergleichs-DNA von Verwandten und Fingerabdrücke der vermissten Person. Spätestens beim Fund der Leiche werden die antemortalen Daten durch die zuständige Dienststelle angefordert. Ein Verdacht bezogen auf die Identität ist hier vorausgesetzt, wie zum Beispiel durch mitgeführte Personaldokumente, das Auffinden in einer angemieteten Wohnung oder Angaben von Zeugen oder Bekannten. Die Erhebung des postmortalen Zahnstatus und der Abgleich von ante- und postmortalen Befunden (Matching) erfolgen durch eine von der Polizei speziell beauftragte Fachperson, z. B. eine/n Zahnmediziner*in oder auch eine/n Rechtsmediziner*in.

Die Akquise vorhandener Daten von vermissten Personen ist laut Literatur ein entscheidender Faktor. Wenn zahnmedizinische Ante-mortem-Daten fehlen oder nur in unzureichender Form vorhanden sind, ist eine Identifizierung anhand des Zahnstatus meist nicht möglich [24], da die ausschlaggebenden Vergleichsdokumente fehlen. Durch die aktuelle Digitalisierung in der Zahnmedizin wird das antemortale Datenmaterial erweitert und kann schneller abgerufen oder übermittelt werden. Moderne dentale Intraoralscanner werden inzwischen bei kieferorthopädischen Planungen und Behandlungen sowie bei prothetischen Behandlungen verwendet, da sie meist komfortabler sind als konventionelle Zahn- und Kieferabdrücke. Diese Scans liefern detailgetreue 3D-Darstellungen der Zähne, nebenbefundlich auch der Gaumenfaltenpaare, die ebenfalls für die Identitätsfeststellung relevant sein können, da sie hochindividuell sind [25–27]. Es ist davon auszugehen, dass die Verwendung der Intraoralscanner zukünftig weiter zunehmen wird [28].

Die Anwendung der forensischen Zahnmedizin bei Identifizierungsprozessen wurde in Deutschland bislang, bezogen auf die polizeilichen Vorgehensweisen, nicht wissenschaftlich aufgearbeitet. Ob und inwieweit ein standardisiertes Vorgehen in der föderalistischen Struktur Deutschlands vorliegt, soll Gegenstand dieser Untersuchung sein. Folglich soll in dieser Studie erstmals die polizeiliche Herangehensweise in den Bundesländern bei der Auffindung von unbekannten Leichen untersucht werden. Welche Identifizierungsmethoden werden angewandt und wie verbreitet ist die Inanspruchnahme forensischer Zahnmedizin? Welche Merkmale werden dabei untersucht und gibt es für die Polizei feste zahnärztliche Ansprechpersonen? Des Weiteren sollte das Potenzial zur Optimierung der Kontaktaufnahme mit forensischen Zahnmediziner*innen herausgearbeitet werden.

Zusätzlich wurden folgende Hypothesen aufgestellt:

1. Das primäre Identifizierungsmerkmal Zahnstatus kommt bundesweit bei der Identifizierung unbekannter Toter zum Einsatz.
2. Die Herangehensweise an die Identifizierung unbekannter Toter ist bundesweit einheitlich.

## Methoden

Der Online-Fragebogen (s. Onlinematerial 1 zu diesem Artikel), bestehend aus 23 Fragen, wurde digital mit der Software SoSci (Version 3.4.03, München, Deutschland) erstellt und nach Prüfung durch die Ethikkommission des Landes Rheinland-Pfalz (2022-16376) an die zuständigen Polizeibehörden gesendet. Dabei wurden mindestens 21 Fragen allen Teilnehmenden angezeigt; in Abhängigkeit von der Beantwortung von Frage 6 (Möglichkeit Ja/Nein) wurde Frage 7 (Antwort: Ja) oder Frage 8 (Antwort: Nein) angezeigt. Frage 18 wurde nur nach Ja-Antwort auf Frage 17 angezeigt.

Der Fragebogen wurde zunächst an die 16 Landesministerien des Inneren versandt und nach dortiger Prüfung und Zustimmung innerhalb des Bundeslandes weitergeleitet. In den Bundesländern wurde der Fragebogen durch eine/n zentrale/n Ansprechpartner*in an die jeweiligen Sachbearbeiter*innen, die sich mit der Bearbeitung von Funden unbekannter Toter im Zuständigkeitsbereich ihrer jeweiligen Dienststelle befassen, verteilt. Jedes Bundesland musste die Befragung folglich zuerst zentral genehmigen und im Anschluss daran an die zuständigen Sachbearbeiter*innen der Dienststellen innerhalb des Bundeslandes weiterleiten.

Der Fragebogen beinhaltete die Abfrage von demografischen Daten wie Alter, Geschlecht, Bundesland und Anzahl der Berufsjahre (Fragen 1–5), sowie die Abfrage nach dem Auffinden von unbekannten Toten in Anzahl und möglicher Dienststellenstatistik (Fragen 5–6). Des Weiteren wurde die Methodik zur Identifizierung abgefragt (Fragen 7–13) und die Rolle der forensischen Zahnmedizin und der Kontaktaufnahme mit den Expert*innen analysiert (Fragen 14–18 und 21). Der letzte Abschnitt des Fragebogens (Fragen 19–20 und 22–23) sollte die Zusammenarbeit mit den zahnmedizinischen Kolleg*innen und Möglichkeiten für eine verbesserte Zusammenarbeit erfragen. In die Auswertung wurden nur vollständig ausgefüllte Fragebögen einbezogen.

Die Daten wurden statistisch aufbereitet und mit SPSS 27 (IBM®, Amonk, NY, USA) ausgewertet. Eine deskriptive Analyse und prozentuale Verteilungsanalyse der Antworten wurden durchgeführt.

## Ergebnis

### Stichprobenbeschreibung

Eine Zustimmung zur Teilnahme erfolgte durch 12 der 16 Bundesländer. Ein weiteres Bundesland musste von der Auswertung ausgeschlossen werden, da ein/e Sachbearbeiter*in den Fragebogen nicht online beantworten konnte. Die Verteilung der Teilnehmenden auf die Bundesländer ist Tab. 1 zu entnehmen. Bei 4 Teilnehmer*innen fehlte die Angabe zum Bundesland ihrer Dienststelle. Insgesamt haben den Fragebogen 85 Beamte*innen erhalten, von denen 77 mit der Befragung begonnen haben. Von diesen haben wiederum 62 den gesamten Fragebogen bearbeitet und konnten in die Auswertung einbezogen werden. Eine Enthaltung war bei allen Fragen möglich.

**Table 1:** 

	Bundesland	Anzahl	Anteil (%)
Gültig	Baden-Württemberg	9	14,5
	Berlin	1	1,6
	Brandenburg	4	6,5
	Bremen	2	3,2
	Hessen	10	16,1
	Mecklenburg-Vorpommern	8	12,9
	Rheinland-Pfalz	16	25,8
	Saarland	1	1,6
	Sachsen-Anhalt	3	4,8
	Schleswig-Holstein	3	4,8
	Thüringen	1	1,6
–	*Gesamt gültig*	*58*	*93,5*
Enthaltungen	–	4	6,5
–	*Gesamt*	*62*	*100,0*

Die Befragten gaben zu 86,5 % (*n* = 53) ein männliches und zu 8,1 % (*n* = 5) ein weibliches Geschlecht an; hier gab es 6,4 % Enthaltungen (*n* = 4). Der Altersdurchschnitt der Teilnehmer*innen betrug 50,5 Jahre (*n* = 58), wobei die jüngste Person 31 und die älteste 61 Jahre alt war. Von den 58 Teilnehmer*innen, die ihre Berufsjahre angegeben haben (4 Enthaltungen), konnte ein Mittelwert von 17 Berufsjahren berechnet werden (Minimum 1 Jahr; Maximum 40 Jahre).

### Auffinden unbekannter Toter

In 83,9 % der Fälle (*n* = 52) wurden den Angaben zufolge bis zu 5 unbekannte Leichen pro Monat im Zuständigkeitsbereich der jeweiligen Dienststelle gefunden, 9,7 % der Befragten (*n* = 6) gaben an, dass es mehr als 5 Funde pro Monat gab (s. Onlinematerial 2, Tab. Z1). 6,5 % der Befragten (*n* = 4) machten keine Angabe.

Eine statistische Erhebung über das Auffinden unbekannter Toter gibt es nach Angaben von 67,7 % (*n* = 42) der Teilnehmenden in ihrer Dienststelle nicht, 14,5 % (*n* = 9) gaben an, dass es eine statistische Erhebung gibt, die restlichen 17,8 % (*n* = 11) machten keine Angaben.

### Methoden zur Identifizierung

Auf die Frage nach den Identifizierungsmethoden, die bei der Entdeckung unbekannter Toter eingesetzt werden (Mehrfachnennungen möglich), gaben 88,7 % die Fingerabdruckanalyse (Daktyloskopie), 87,1 % die forensische Zahnmedizin und 91,9 % die DNA-Analyse als Methode nach INTERPOL-Standard an (Tab. 2). Folglich werden in der Praxis meist Kombinationen der 3 primären Merkmale DNA, Fingerabdruck und Zahnstatus zur Identifizierung verwendet. Darüber hinaus gaben 87,1 % der Befragten die Untersuchung sekundärer Merkmale wie „Kleidung, Tätowierungen etc.“ als Identifizierungsmethoden an. Insgesamt gaben 72,6 % an, dass bei der Identifizierung verschiedene Methoden kombiniert werden (s. Onlinematerial 2, Tab. Z2).

**Table 2:** 

Identifizierungsmethode	Anzahl der Angaben	Anteil (in %)	Anzahl ohne Angabe	Anteil (in %)	Gesamt
Fingerabdruckanalyse	55	88,7	7	11,3	62
Forensische Zahnmedizin	54	87,1	8	12,9	62
DNA-Analyse	57	91,9	5	8,1	62
Sekundäre Merkmale	54	87,1	8	12,9	62

**Figure 1:**
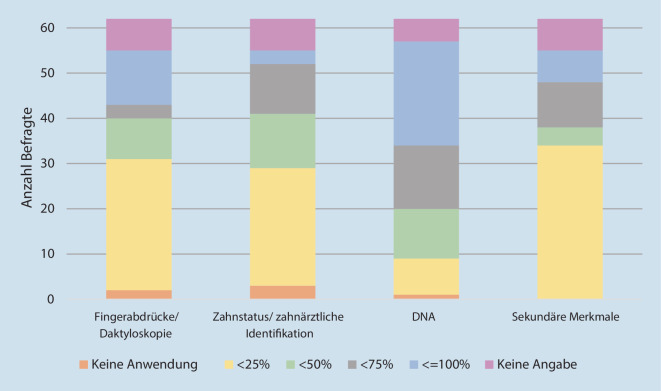

Der prozentuale Anteil der jeweiligen Methoden Fingerabdrücke/Daktyloskopie, Zahnstatus/zahnärztliche Identifizierung, DNA und sekundäre Merkmale wurde von den Beamten*innen in Quartilen geschätzt: 0 %/< 25 %/< 50 %/< 75 %/≤ 100 % (Abb. 1). Am häufigsten kommt demnach die DNA-Analyse zum Einsatz, hier wurde ein Anteil von > 50 % durch 59,7 % der Befragten angegeben (Untersuchung sekundärer Merkmale: 27,4 %, Fingerabdruckanalyse: 24,2 %, forensische Zahnmedizin 22,6 %).

Von den Beamte*innen gaben 72,6 % an, dass Sie, um noch erfolgreicher zu sein, eine Kombination der primären Identifizierungsmethoden anwenden. Dabei wurde mit 37,1 % (*n* = 23) am häufigsten die Kombination aus DNA-Analyse und Zahnstatus genannt (Tab. 3).

**Table 3:** 

Kombinationen	Anzahl der Angaben	Anteil (%)
Dental + DNA	23	37,1
Fingerabdruck + Dental + DNA	17	27,4
Fingerabdruck + DNA	13	21,0
Andere	4	6,5
Keine Angabe	5	8,1
Gesamt	62	100,0

### Einbeziehung von forensischer Zahnmedizin

62,9 % der Befragten stimmten zu, dass die zahnärztliche Identifizierung „oft“ angewendet wird. Von den vorgegebenen möglichen Begründungen (Mehrfachantworten möglich) wurde am häufigsten ausgewählt: „weil eine eindeutige Identifikation möglich ist (post-mortem Zahnstatus entspricht genau ante mortem Zahnstatus)“. Umgekehrt stimmten 37,1 % der Befragten nicht zu, dass die zahnärztliche Identifizierung „oft“ angewendet wird. Die Mehrheit (30,6 %) der Befragten gab dafür „Sonstiges“ als Grund an, machte aber im Freitext keine spezifischen Angaben. 12,9 % kreuzten an: „weil es sich um eine arbeitsaufwendige Methode handelt (Anfordern von antemortem Zahnstatus)“, und weitere 11,3 % kreuzten an: „weil die Mithilfe von externen Institutionen notwendig ist (Zahnärzte/-innen)“.

Beamten*innen aus Dienststellen mit einer statistischen Erhebung wurden gefragt, welcher Anteil der unbekannten Toten mittels Zahnstatus identifiziert wird. Die angegebenen Werte lagen zwischen 1,6 % und 8,1 %, wobei in 61 % dieser Fälle die Prozentwerte unter 5 % lagen. Die Teilnehmer*innen, in deren Dienststellen es keine statistische Erhebung gab, wurden gebeten den Anteil zu schätzen. Dabei wurden Werte zwischen 1,6 % und 11,3 % angegeben. 22,6 % dieser Befragten (*n* = 14) gaben hierbei Prozentwerte von unter 5 % an.

Als Identifizierungsmethoden, die im Rahmen der forensischen Zahnmedizin angewendet werden, gaben die Befragten mit 54,8 % den Vergleich von Post-mortem- mit Ante-mortem-Zahnstatus mithilfe des Dental Profiling an (Untersuchungen der Zähne, Knochenstruktur, Mundhöhle, Weichteile, um ein Profil zu erstellen, Röntgenbilder, Zahnstatus). 21,0 % wählten die „DNA-Gewinnung aus einem Zahn mit nachfolgender DNA-Analyse“ aus. Nur wenige kreuzten odontometrische Geschlechtsbestimmung (*n* = 1), Altersdiagnostik (*n* = 3) und das Auslesen von Medizinprodukten (z. B. Implantat-Identifizierungsnummer; *n* = 1) an. Eine Person wählte keine der vorgegebenen Methoden aus und gab im Freitextfeld „hier nicht bekannt“ an.

Auf die Frage, wie der postmortale Zahnstatus in der eigenen Organisationseinheit erhoben wird (Mehrfachantworten möglich), kreuzten 90,3 % (*n* = 56) „Rechtsmedizin (Obduktion)“ an, 21 % der Befragten (*n* = 13) wählten „Externe Spezialisten (z. B. spezialisierte Zahnärzte/-innen)“. Weiterhin kreuzten 6,5 % (*n* = 4) „Andere“ an und gaben im Freitextfeld jeweils zusätzlich Folgendes an: Zahnärzt*innen des Bundeskriminalamtes (*n* = 1), Identifizierungskommission (IDKO; *n* = 1), Kassenzahnärztliche Vereinigung (*n* = 1). Eine Angabe lautete: „selten Zahnärzte, die bei der Obduktion hinzugezogen werden“.

Die Kontaktaufnahme zu Zahnärzt*innen zwecks Abgleich post mortem erfolgt laut den Angaben der Befragten zu 25,8 % (*n* = 16) über digitale Medien, zu 19,4 % (*n* = 12) über Printmedien (z. B. Zeitschriften wie die *Zahnärztlichen Mitteilungen*) und zu 11,3 % (*n* = 7) über Fachkreise (z. B. Arbeitskreis für forensische Odontostomatologie) an. Das Freitextfeld wurde von 51,6 % (*n* = 32) genutzt, hier wurden Direktkontakte (*n* = 15), die Rechtsmedizin (*n* = 6) und versicherungstechnische Institutionen (*n* = 6) angegeben.

Auf die Frage, ob eine ständige zahnärztliche Ansprechperson zur Verfügung steht, antworteten 69,4 % (*n* = 43) mit „Nein“, 7 Personen machten keine Angaben dazu. Von den 19,4 % (*n* = 12) Beamten*innen, die diese Frage mit „Ja“ beantworteten, wurden als Ansprechpersonen „Allgemeine/r Zahnarzt/-ärztin“ (*n* = 7), „Forensische/r Odonto-Stomatologe/-in“ (*n* = 4) oder „Fachzahnarzt/-ärztin für Kieferorthopädie“ (*n* = 2) angekreuzt. 22,6 % (*n* = 14) der Befragten kreuzten „Andere“ an. Im dazugehörigen Freitextfeld wurden ein „Facharzt für Prothetik“ (*n* = 1), ein „Facharzt für Zahnheilkunde“ (*n* = 1) und ein „forensischer Pathologe“ (*n* = 1) angegeben. 2 weitere Eintragungen lauteten „nicht bekannt“.

### Zusammenarbeit mit Zahnärzt*innen

Auf die Frage, wie oft eine Rückmeldung der kontaktierten Zahnmediziner*innen erfolgt, gaben die meisten Beamten*innen „immer“ an (40,3 %; *n* = 25; Tab. 4). 4,8 % (*n* = 3) kreuzten „nie“ an. Auf die Frage, von welchen Faktoren die zahnärztliche Rückmeldung nach Meinung der Befragten abhänge, wählten die meisten als Antwort (Mehrfachantworten möglich): „Gut geführte Patientenakten mit vollständigem Zahnstatus (ante-mortem)“ (61,3 %; *n* = 38), gefolgt von „Eindeutiger Zahnstatus post-mortem“ (56,5 %; *n* = 35) und „Moderne Praxen (Digitalisierung)“ (27,4 %; *n* = 17). Eine saisonale Abhängigkeit (Sommer‑/Urlaubszeit) wurde nur von 6,5 % (*n* = 4) der Befragten als Grund angekreuzt. Allerdings kreuzten 8,1 % (*n* = 5) als Antwortmöglichkeit an, dass sich die Mundgesundheit der Bevölkerung verbessert habe und somit weniger zahnärztliche Behandlungen (weniger Zahnersatz, weniger Röntgenbilder) der Grund für die fehlende Rückmeldung sei. Im Freitextfeld wurden weitere wahrscheinliche Gründe jeweils von 1 Person angegeben: mangelndes Interesse an der forensischen Zahnmedizin, ein hoher Aufwand beim Vergleich des ante- und postmortalen Zahnstatus und mangelnde Vergütung.

**Table 4:** 

Antwort	Anzahl	Anteil (%)
Immer	25	40,3
Oft	10	16,1
Manchmal	3	4,8
Selten	3	4,8
Nie	3	4,8
Keine Angabe	18	29
Gesamt	62	100,0

Die Frage, ob eine digitale Plattform zur Kontaktaufnahme mit Zahnärzt*innen hilfreich wäre, wurde von der Mehrheit (56,5 %; *n* = 35) mit „sehr hilfreich“ beantwortet (s. Onlinematerial 2, Tab. Z3). Nur eine Person kreuzte an, dass diese „gar nicht hilfreich“ sei. Die Mehrheit der Befragten (62,9 %; *n* = 39) gab an, dass ein „zentraler Ansprechpartner pro Bundesland (Forensische/r Odontostomatologe/in)“ die primäre Anwendung der zahnärztlichen Identifizierung beim Fund eines unbekannten Toten erleichtern würde (s. Onlinematerial 2, Tab. Z4). Für 35,5 % (*n* = 22) wäre ein Register für zahnärztliche Befunde hilfreich, für 12,9 % (*n* = 8) ein Datenblatt im Sinne einer standardisierten Checkliste für das Vorgehen der zahnmedizinischen Identifizierung.

## Diskussion

In dieser Studie konnte die polizeiliche Vorgehensweise auf Ebene der teilnehmenden Bundesländer bei der Bearbeitung von Funden unbekannter Toter analysiert werden. Dabei wurden die Anwendung der forensischen Zahnmedizin und die Bewertung der Methodik aus polizeilicher Sicht sowie die Bewertung des Zahnstatus als primäres Identifizierungsmerkmal evaluiert und eine mögliche Optimierung in der Zusammenarbeit zwischen Polizei und Zahnmedizin untersucht.

### Stichprobe und Methodik

Von den 16 Bundesländern lag die Zustimmung von 12 zur Teilnahme vor, was die Aussage über eine bundesweite Vorgehensweise im Kontext der Identifizierungsweise beim Auffinden von unbekannten Toten verhindert. Da die Steuerung der Befragung über bundeslandspezifische Ansprechpartner*innen erfolgte, ist die Gesamtzahl der Beamte*innen, die pro Bundesland mit den Funden unbekannter Toter betraut sind, nicht nachzuvollziehen. Es kann jedoch aufgrund der zentralen Steuerung davon auszugegangen werden, dass alle Befragten mit der Thematik sicher vertraut sind und sich mit diesen Vorgängen als Sachbearbeiter*innen der entsprechenden Bundesländer befassen. Folglich sind die Antworten von fachlich versierten Beamten*innen als repräsentativ zu werten. Von 85 angesteuerten Befragten ist die Rücklaufquote mit 72,9 % (*n* = 62) als hoch einzuschätzen und somit als repräsentativ einzuordnen.

Die Methodik dieser Studie setzte eine Online-Durchführung voraus und beinhaltete keine alternative Möglichkeit der Beantwortung, was zum Ausschluss eines weiteren Bundeslandes führte. Für weiterführende Befragungsprojekte wäre die Möglichkeit eines alternativen, analogen Fragebogens in Betracht zu ziehen. Der hohe Anteil von männlichen Teilnehmenden (86,5 %) spiegelt die aktuelle Geschlechterverteilung der Polizei in den Landesbehörden wider [29]. Das Durchschnittsalter (50,5 Jahre) und die durchschnittliche Anzahl der Berufsjahre (17 Jahre) lassen die Vermutung zu, dass die Beamte*innen, die mit der Bearbeitung von Funden unbekannter Toter betraut sind, ein hohes Maß an Berufserfahrungen aufweisen.

Es können die folgenden Schlussfolgerungen hinsichtlich der anfangs aufgestellten Hypothesen gezogen werden: Die erste Hypothese, dass das primäre Identifizierungsmerkmal Zahnstatus bundesweit bei der Identifizierung unbekannter Toter zum Einsatz kommt, kann nicht bestätigt werden, da nicht alle Bundesländer teilnahmen und auch bei den teilnehmenden Dienststellen divergierende Ergebnisse festgestellt wurden. Die zweite Hypothese, dass die Herangehensweise an die Identifizierung unbekannter Toter bundesweit einheitlich ist, muss damit auch verworfen werden. Die individuelle Bearbeitung von Fällen obliegt den zuständigen Dienststellen und weist große Unterschiede bezogen auf den Einsatz von zahnärztlicher Identifizierung auf.

### Auffindung unbekannter Toter und Methoden zur Identifizierung

Da 67,7 % der Befragten angaben, dass keine statistischen Erhebungen in ihrem Dezernat dazu bestehen, wie viele Fälle von unbekannten Toten pro Monat bearbeitet werden, sind die gemachten Angaben der Mehrheit subjektiver Natur und lassen die Vermutung aufkommen, dass eine breite Varianz bestehen könnte. Für eine bundesweite Zusammenarbeit der Länder könnten eine statistische Erhebung der Fälle und eine übergreifende Einsicht in die Fallakten von hohem Wert sein, um Ressourcen zu schonen und Identifizierungen schneller durchführen zu können.

Die Identifizierungsmethoden DNA- und Fingerabdruckanalyse sowie forensische Zahnmedizin finden dienststellenspezifisch in unterschiedlichem Maße Anwendung. Die DNA-Analyse wird deutlich häufiger angewendet als die forensische Zahnmedizin (Anwendung in über 50 % der Fälle bei DNA-Analyse: 59,7 %, bei forensischer Zahnmedizin 22,6 %). Hier wird ein großes Potenzial zum Ausbau der forensischen Zahnmedizin erkennbar, wenn der in der Literatur beschriebene Einsatz und zielführende Erfolg der forensischen Zahnmedizin bei der Analyse von Massenkatastrophen, wie zum Beispiel dem Tsunami von 2004, betrachtet wird [4]. In einer Studie von Obafunwa et al., in der ein Flugzeugunglück mit 152 Opfern auf Lagos von 2012 analysiert wurde, wird allerdings ebenfalls ein geringer Anteil an forensischer Zahnmedizin im Identifizierungsprozess beschrieben. Hier wurden nur 10 % über den Zahnstatus allein identifiziert [30], wohingegen die Kombination mit der DNA-Analyse mit 97,4 % zum Erfolg geführt hat. Dies deckt sich mit den Ergebnissen unserer Studie, bezogen auf die präferierte Kombination der primären Marker DNA und Zahnstatus bei 37,1 % der Befragten.

Zu diskutieren ist in diesem Zusammenhang, dass die forensische Zahnmedizin bei vorliegenden Vergleichsdaten im Gegensatz zur DNA-Analyse sehr schnell und kostengünstig in der Anwendung sein kann. Dass die Beauftragung der forensischen Zahnmedizin laut unseren Ergebnissen deutlich seltener stattfindet, kann verschiedene Gründe haben, denkbar sind schlechte Erfahrungswerte (wie beispielsweise durch mangelnde Verfügbarkeit), Unkenntnis über die Methodik oder auch ein fehlendes Netzwerk mit Zahnmediziner*innen, die postmortale Befunde am Leichnam erheben. Die Diversität in der Herangehensweise innerhalb der befragten Bundesländer lässt keinen richtigen Vergleich unter den Bundesländern zu. Eine statistische Erfassung und eine zumindest bundeslandinterne Vereinheitlichung der Prozesse könnten zu schnelleren Ergebnissen bei der Identifizierung und mehr Wirtschaftlichkeit beitragen. Eine bundesweite Vereinheitlichung scheint in diesem Kontext aufgrund der föderalistischen Struktur Deutschlands als kritisch in der Umsetzbarkeit.

### Einbeziehung von forensischer Zahnmedizin

Eine grundlegende Voraussetzung für die Anwendung von forensischer Zahnmedizin im Identifizierungsprozess ist eine gute zahnmedizinische Versorgung der Bevölkerung, damit antemortale Daten verfügbar sind, die im Schadensereignis für einen postmortalen Vergleich herangezogen werden können. Die zahnärztliche Versorgung, im Hinblick auf kassenzahnärztliche Leistungspositionen wie die Jahreskontrolle und Subvention von zahnmedizinischer Grundversorgung, ist in Deutschland auf Bundesebene geregelt. Auf internationaler Ebene variiert die Versorgung länderabhängig und in Entwicklungsländern ist sie zum Teil gar nicht vorhanden [21]. In der Literatur wurde deutlich herausgearbeitet, dass die zahnärztlichen antemortalen Aufzeichnungen von entscheidender Bedeutung bei der Identifizierung unbekannter Toter sind, diese aber oft vollständig fehlen und nicht akquiriert werden können [24, 31]. In einer Studie von Petju et al. [31] wurde eruiert, dass, sollten zahnmedizinische Unterlagen vorhanden sein, eine signifikant schnellere Identifizierung durchgeführt werden kann als mit anderen Methoden wie der DNA-Analyse (*p* < 0,01), was sich mit der Bewertung vonseiten der Beamten*innen deckt, die die Zahnmedizin insgesamt als gut geeignete Methode beim Abgleich von ante- und postmortalen Befunden wertet.

In den befragten Bundesländern gab die Mehrheit mit 62,9 % der Beamte*innen an, dass die zahnärztliche Identifizierung „oft“ Anwendung findet, jedoch konnte der in der Literatur beschriebene Aspekt der problematischen antemortalen Datenakquise als einer von 2 Gründen, die dagegen sprechen, bei 12,9 % festgestellt werden. Weitere 11,3 % gaben an, dass die notwendige Zuhilfenahme von externen Institutionen gegen eine häufige Anwendung von forensischer Zahnmedizin spricht.

Wenn keine antemortalen Vergleichsdaten vorliegen, können in der dentalen Forensik auch andere Untersuchungen wie die Geschlechtsbestimmung, die dentale Altersdiagnostik [32] oder das Auslesen von Medizinprodukten wie Implantaten mit Kennnummer durchgeführt werden. Diese Aspekte wurden in unserer Studie aufgegriffen, gehören jedoch laut unserer Ergebnisse nicht zum gängigen Repertoire der Beauftragung durch die Beamten*innen. Sie sind nicht in allen Dienststellen bekannt. Ansprechpartner*innen für die forensische Zahnmedizin stehen bei 69,4 % der Befragten nicht permanent zur Verfügung und die postmortale Erhebung des Zahnstatus erfolgt laut 90,3 % der Befragten in der Rechtsmedizin. Folglich ist ein direkter Kontakt mit Zahnmedizinern, insbesondere forensisch weitergebildeten Experten*innen, für die Mehrheit der polizeilichen Sachbearbeiter*innen nicht gegeben, was kritisch zu bewerten ist, da in der Literatur nachgewiesen werden konnte, dass ein höheres Expertenlevel mit besseren Ergebnissen im Identifikationsprozess einhergeht [33].

Zur Kontaktaufnahme mit forensischen Zahnärzt*innen nehmen die Beamte*innen den Weg über digitale Medien, Printmedien und Fachkreise, was für sie schwierig sein kann, wenn keine zahnmedizinischen Vorkenntnisse vorliegen. Es lässt sich vermuten, dass die Beamten*innen insgesamt bisher noch wenig Bezug zur forensischen Zahnmedizin haben. Bundesweite Fort- und Weiterbildungen zur Identifizierung unbekannter Toter mittels forensischer Zahnmedizin, die auch die Vielfalt der einzelnen Untersuchungsmethoden in diesem Gebiet aufzeigen, könnten dazu beitragen, dass die forensische Zahnmedizin in Deutschland zukünftig noch stärkere Verbreitung findet.

### Zusammenarbeit mit Zahnärzt*innen

In der Zusammenarbeit mit den Zahnmedizinern gibt es grundlegend 2 Kontaktpunkte für die Polizeikräfte: 1) die postmortale Befundung am Leichnam und 2) die Akquise antemortaler Daten von Zahnärzt*innen in der Niederlassung oder in Universitäten, die den/die Verstorbene/n zu Lebzeiten behandelt haben. Die Ergebnisse zeigen, dass die Beamte*innen bei Anfragen zu antemortalen Daten oft keine Antwort erhielten (nur 40,3 % kreuzten „immer“ an). Bei einer polizeilich geforderten Rückmeldung scheint das ungewöhnlich.

Die Befragten gaben mit 61,3 % am häufigsten an, dass eine Antwort aus ihrer Sicht von „gut geführten Patientenakten mit vollständigem Zahnstatus (ante-mortem)“ abhängig ist. Dieses Ergebnis muss im Hinblick auf die Regelung gemäß § 630f BGB kritisch diskutiert werden, da seit dem 26.02.2013 im BGB festgelegt wurde, dass sowohl für vertragszahnärztliche als auch für privatzahnärztliche Tätigkeiten eine Aufbewahrungspflicht der Behandlungsunterlagen von 10 Jahren gilt [34]. Ausgenommen davon sind zahnärztliche Modelle, die mitunter kürzeren Aufbewahrungspflichten unterliegen. Folglich wäre eine fehlende Dokumentation trotz Behandlung oder eine Entsorgung vor Ablauf der Aufbewahrungsfrist strafbar.

Diese mehrheitliche Antwort vonseiten der Beamte*innen könnte jedoch auch auf gänzlich fehlende antemortale Zahndaten zurückführbar sein, da die unbekannten tot aufgefundenen Menschen in vielen Fällen zu Lebzeiten einen ungeregelten Lebensstil hatten und daher auch die Vorstellung bei Zahnmediziner*innen ausblieb. Es wären auch keine zahnärztlichen Befunde in den Praxen zu akquirieren, wenn die Behandlung mind. 10 Jahre zurückliegt. Dazu passt auch die zweithäufigste Antwort mit 56,5 % („eindeutiger Zahnstatus post-mortem“), da beim Auffinden von zahnlosen Mundsituationen oder bei nur wenig charakteristischen zahnmedizinischen, mundhygieneinsuffizienten Funden der Verdacht nahe liegt, dass die/der Verstorbene/r keinen Hauszahnarzt aufgesucht hat und folglich Anfragen ins „Leere“ laufen.

Die logisch erscheinende saisonale Abhängigkeit der Antworten von Sommer- und Urlaubszeiten wurde hingegen nur von 6,5 % der Befragten angekreuzt. Dieses Phänomen konnte sowohl beim Einsatz in der Ahrtalkatastrophe zu Beginn der Ferienzeit als auch bei den Anschlägen auf dem Breitscheidplatz zur Weihnachtszeit beobachtet werden, da eine teilweise erschwerte Erreichbarkeit der Zahnärzt*innen mitunter urlaubsbedingt war.

Zusätzlich wurde die Modernität der Praxen von 27,4 % der Befragten als beeinflussender Faktor für Rückmeldungen angekreuzt. Eine moderne Ausstattung der Zahnarztpraxis ermöglicht eine schnelle und effiziente Abrufbarkeit von Daten, wie zum Beispiel von Röntgenbildern, was im Rahmen von Massenkatastrophen mit Zeitersparnis im Identifizierungsprozess einhergehen würde [10].

Die Mehrheit der Befragten bewertet die Schaffung einer digitalen Plattform als „sehr hilfreich“ (56,5 %), ein zentraler Ansprechpartner pro Bundesland (forensischer/r Odontostomatologe/in) wurde von 62,9 % der Beamte*innen befürwortet Dieses Ergebnis zeigt auf, dass vonseiten der Polizeikräfte eine Optimierung in der Interaktion mit den Zahnärzt*innen gewünscht ist und die Kontaktaufnahme eine Hürde zu sein scheint. Für 35,5 % der Befragten wäre ein Register wünschenswert. Polizeikräfte kennen solche „Register“ im Sinne einer zentralen Datenbank wie bei gespeicherten Fingerabdrücken im AFIS (Automatisiertes Fingerabdruckidentifizierungssystem). Dieses Matching-Tool wurde in den letzten Jahrzehnten konstant weiterentwickelt, um eine Verfeinerung im Abgleich zu erzielen und dadurch die polizeiliche Arbeit zu unterstützen [35, 36]. Ein entsprechendes Tool für die Zahnmedizin steht auf Länderebene bisher nicht zur Verfügung. Das bei Massenkatastrophen vom Bundeskriminalamt zum Einsatz kommende Programm PlassData könnte einen solchen Ansatz für die Länderpolizei darstellen, setzt aber Training im Umgang mit ante- und postmortalen Daten voraus. Bei guter Qualität der Daten könnte ein teilautomatisiertes Matching von Vermisstenfällen stattfinden, was jedoch für die abschließende Identifizierung einen forensischen Experten für alle Teilschritte voraussetzen würde [37].

Insgesamt lässt sich bezogen auf die Zusammenarbeit mit den Zahnmedizinern vermuten, dass eine bundesweite Sensibilisierung hinsichtlich der zur Verfügungstellung von antemortalen Daten sowie ein flächendeckendes System an geschulten zahnärztlich forensischen Ansprechpartner*innen eine Verbesserung der interdisziplinären Zusammenarbeit mit der Polizei auf Länderebene mit sich bringen könnte.

## Fazit

Die vorliegende Studie konnte die Vielfältigkeit der Herangehensweise der Länderpolizei bei der Identifizierung unbekannter Toter in den einzelnen Dienststellen aufzeigen. Während bei allen Teilnehmenden grundlegendes Wissen über die Bedeutung des Zahnstatus als primärer Marker vorhanden war, offenbarten sich teilweise Lücken im detaillierten Verständnis der spezifischen Teilaspekte, die in Identifizierungsprozessen zum Tragen kommen können. Strukturierte bundesweite Fort- und Weiterbildungen für Polizeikräfte könnten hier Abhilfe schaffen. Dass auch nach erwiesenen Erfolgen in Großschadenslagen noch immer eine Tendenz zur kosten- und zeitintensiveren DNA-Analyse in der Beauftragung besteht, scheint häufig mit fehlenden zahnmedizinischen Ansprechpartner*innen vor Ort einherzugehen, sowie den Limitationen bei der Akquise antemortaler Daten. Die Datenakquise könnte aber mit der zunehmenden Digitalisierung in der zahnärztlichen Niederlassung und im universitären Umfeld zukünftig erleichtert werden. Forschungsansätze im zahnmedizinischen Bereich zu weiteren individuellen Merkmalen in der Mundhöhle, zusätzlichen Identifizierungsmethoden sowie KI-basiertem Matching könnten zukünftig zur Verbreitung und zum Erfolg der forensischen Zahnmedizin beitragen.

### Supplementary Information






## References

[1] Sweet D (2010). INTERPOL DVI best-practice standards—An overview. Forensic Sci Int.

[2] Interpol (2023) Disaster Victim Identification (DVI). https://www.interpol.int/How-we-work/Forensics/Disaster-Victim-Identification-DVI. Zugegriffen: 2023-04-06

[3] Arrighi E, Charlot AM (2020). Identifying terrorist attack victims. Forensic Sci Res.

[4] James H (2005). Thai tsunami victim identification overview to date. J Forensic Odontostomatol.

[5] Schuller-Götzburg P, Suchanek J (2007). Forensic odontologists successfully identify tsunami victims in Phuket, Thailand. Forensic Sci Int.

[6] Schuller-Götzburg P (2016). Forensisch-odontologische Identifizierung der Opfer des Flugunfalls Zell am See. Rechtsmedizin.

[7] De Valck E (2006). Major incident response: collecting ante-mortem data. Forensic Sci Int.

[8] Komuro T, Tsutsumi H, Izawa H (2019). Social contribution of forensic odontology in Japan. Jpn Dent Sci Rev.

[9] Schuller-Götzburg P (2007). Dental Identification of Tsunami Victims in Phuket, Thailand. Dentalna identifikacija žrtava cunamija u Phuketu, Tajland. Acta Stomatol Croat.

[10] Forrest A (2019). Forensic odontology in DVI: current practice and recent advances. Forensic Sci Res.

[11] Jordan RA, Bodechtel C, Hertrampf K (2014). The Fifth German Oral Health Study (Funfte Deutsche Mundgesundheitsstudie, DMS V)—rationale, design, and methods. BMC Oral Health.

[12] Martin-de-Las-Heras S, Valenzuela A, Luna Jde D, Bravo M (2010). The utility of dental patterns in forensic dentistry. Forensic Sci Int.

[13] Bathala LR, Rachuri NK, Rayapati SR, Kondaka S (2016). Prosthodontics an ‚arsenal‘ in forensic dentistry. J Forensic Dent Sci.

[14] Gosavi S, Gosavi S (2012). Forensic odontology: a prosthodontic view. J Forensic Dent Sci.

[15] Mishra SK, Mahajan H, Sakorikar R, Jain A (2014). Role of prosthodontist in forensic odontology. A literature review. J Forensic Dent Sci.

[16] Manigandan T, Sumathy C, Elumalai M, Sathasivasubramanian S, Kannan A (2015). Forensic radiology in dentistry. J Pharm Bioallied Sci.

[17] Heinrich A, Guttler F, Wendt S (2018). Forensic odontology: automatic identification of persons comparing antemortem and postmortem panoramic radiographs using computer vision. Rofo.

[18] Heinrich A, Guttler FV, Schenkl S, Wagner R, Teichgraber UK (2020). Automatic human identification based on dental X-ray radiographs using computer vision. Sci Rep.

[19] Baelum V (2010). What is an appropriate caries diagnosis?. Acta Odontol Scand.

[20] Chen P, Nikoyan L (2021). Guided implant surgery: a technique whose time has come. Dent Clin North Am.

[21] Kernen F, Kramer J, Wanner L, Wismeijer D, Nelson K, Flugge T (2020). A review of virtual planning software for guided implant surgery—data import and visualization, drill guide design and manufacturing. BMC Oral Health.

[22] Toupenay S, Cheikh AB, Ludes B, Felizardo R (2020). Forensic odontology identification response to terrorist attacks in Paris November 2015. Forensic Sci Res.

[23] France Interministerial Directions on the Care of Victims of Terrorism Acts (2019) Instruction interministerielle relative à la prise en charge des victimes d’actes de terrorisme. http://circulaires.legifrance.gouv.fr/pdf/2019/03/cir_44445.pdf. Zugegriffen: 14. Aug. 2023

[24] Prajapati G, Sarode SC, Sarode GS, Shelke P, Awan KH, Patil S (2018). Role of forensic odontology in the identification of victims of major mass disasters across the world: A systematic review. PLoS ONE.

[25] Burzynski JA, Firestone AR, Beck FM, Fields HW, Deguchi T (2018). Comparison of digital intraoral scanners and alginate impressions: Time and patient satisfaction. Am J Orthod Dentofacial Orthop.

[26] Winkler J, Gkantidis N (2021). Intraoral scanners for capturing the palate and its relation to the dentition. Sci Rep.

[27] Bjelopavlovic M, Degering D, Lehmann KM, Thiem DGE, Hardt J, Petrowski K (2023). Forensic identification: dental scan data sets of the palatal fold pairs as an individual feature in a longitudinal cohort study. Int J Environ Res Public Health.

[28] Schott TC, Arsalan R, Weimer K (2019). Students’ perspectives on the use of digital versus conventional dental impression techniques in orthodontics. BMC Med Educ.

[29] Bundesamt PNNS (2020) Zahl der Polizeianwärterinnen und -anwärter seit 2010 mehr als verdoppelt. https://www.destatis.de/DE/Presse/Pressemitteilungen/2020/09/PD20_N057_742.html. Zugegriffen: 14. Aug. 2023

[30] Obafunwa JO, Ogunbanjo VO, Ogunbanjo OB, Soyemi SS, Faduyile FA (2015). Forensic odontological observations in the victims of DANA air crash. Pan Afr Med J.

[31] Petju M, Suteerayongprasert A, Thongpud R, Hassiri K (2007). Importance of dental records for victim identification following the Indian Ocean tsunami disaster in Thailand. Public Health.

[32] Krishan K, Kanchan T, Garg AK (2015). Dental evidence in forensic identification—an overview, methodology and present status. Open Dent J.

[33] Pinchi V, Norelli GA, Caputi F, Fassina G, Pradella F, Vincenti C (2012). Dental identification by comparison of antemortem and postmortem dental radiographs: influence of operator qualifications and cognitive bias. Forensic Sci Int.

[34] Justiz Bd Bürgerliches Gesetzbuch (BGB) § 630f Dokumentation der Behandlung. https://www.gesetze-im-internet.de/bgb/__630f.html. Zugegriffen: 14. Aug. 2023

[35] Neumann C, Armstrong DE, Wu T (2016). Determination of AFIS ‚sufficiency‘ in friction ridge examination. Forensic Sci Int.

[36] Hefetz I, Liptz Y, Oz K (2021). The benefit of AFIS searches of lateral palm and non-distal phalanges prints in criminal investigation. Forensic Sci Int.

[37] Kieser JA, Laing W, Herbison P (2006). Lessons learned from large-scale comparative dental analysis following the South Asian tsunami of 2004. J Forensic Sci.

