# Supplementary material for: Forensic dentistry for identity verification. A survey at the state police level
Source: Bundesgesundheitsblatt Gesundheitsforschung Gesundheitsschutz. 2023 Sep 27;66(11):1268–76. [Article in German] doi: 10.1007/s00103-023-03769-2 (PMC10622376; doi:10.1007/s00103-023-03769-2)
Supplement: Supplementary file 1 [file 103_2023_3769_MOESM1_ESM.pdf]

Onlinematerial 1: Liste der im Onlinefragebogen enthaltenen Fragen

1. **Wie alt sind Sie?**

2. **Bitte geben Sie ihr Geschlecht an:**

   ☐ *Weiblich*
   ☐ *Männlich*
   ☐ *Divers*

3. **In welchem Bundesland arbeiten Sie?**

4. **Über wie viele Berufsjahre in der Bearbeitung von Todesermittlungssachen, auch bei unbekannten Toten, verfügen Sie?**

5. **Wie häufig kommt es zum Fund eines unbekannten Leichnams pro Monat? (Durchschnitt in den letzten 10 Jahren)**

   ☐ *< 5 Funde pro Monat*
   ☐ *> 5 Funde pro Monat*
   ☐ *> 10 Funde pro Monat*
   Eigene Angabe: *Funde pro Monat*

6. **Gibt es in Ihrem Bundesland statistische Erhebungen hinsichtlich erfolgreicher Identifikation von unbekannten Toten?**

   ☐ *Ja* *-> Bei Auswahl „Ja“ folgt Frage 7 für die Befragten, Frage 8 entfällt*
   ☐ *Nein* *-> Bei Auswahl „Nein“ folgt direkt Frage 8 für die Befragten, Frage 7 entfällt*

7. **Wie viel Prozent der unbekannten Toten werden primär mit Hilfe der zahnärztlichen Identifikation bestimmt?**

   % werden mit Hilfe der dentalen Identifikation bestimmt.

8. **Wie schätzen Sie den prozentualen Anteil ein, der primär mit Hilfe der zahnärztlichen Identifikation bestimmt wird?**

%

9. **Bei der Auffindesituation eines unbekannten Leichnams werden folgende Identifikationsmechanismen angewendet:**

   Mehrfachnennung möglich

   ☐ Fingerabdrücke/Daktyloskopie
   ☐ Zahnstatus/zahnärztliche Identifikation
   ☐ DNA
   ☐ Sekundäre Merkmale (Kleidung, Tätowierungen etc.)
   Andere:

10. **Nennen Sie den prozentualen Anteil der Methoden, die zur Identifizierung angewendet werden?**

	**0%**	**<25%**	**<50%**	**<75%**	**<=100%**
**Fingerabdrücke/Daktyloskopie**	☐	☐	☐	☐	☐
**Zahnstatus/zahnärztliche Identifikation**	☐	☐	☐	☐	☐
**DNA**	☐	☐	☐	☐	☐
**Sekundäre Merkmale (Kleidung, Tätowierungen etc.)**	☐	☐	☐	☐	☐

11. Welche der Methoden eignen sich am besten zur Identifikation?

1 = nicht ausreichend, 2 = ausreichend, 3 = befriedigend, 4 = gut, 5 = sehr gut

	1	2	3	4	5
**Fingerabdrücke/Daktyloskopie**	☐	☐	☐	☐	☐
**Zahnstatus/zahnärztliche Identifikation**	☐	☐	☐	☐	☐
**DNA**	☐	☐	☐	☐	☐
**Sekundäre Merkmale (Kleidung, Täto-wierungen etc.)**	☐	☐	☐	☐	☐

12. Ist eine Kombination der primären Identifizierungsmethoden, um noch erfolgreicher zu sein, Standard?

☐ Ja
☐ Nein

13. Welche Methoden werden bevorzugt miteinander kombiniert?

☐ Fingerabdrücke + Zahnstatus + DNA
☐ Fingerabdrücke + Zahnstatus
☐ Fingerabdrücke + DNA
☐ Zahnstatus + DNA
☐ Andere:

14. Die zahnärztliche Identifikation ist eine gängige Methode zur Identifizierung von unbekannten Toten. Wird sie oft angewandt?

Ja oder Nein wählen + Begründung als Mehrfachnennung

☐ ***Ja, weil…***

- ☐ *... eine eindeutige Identifikation möglich ist (post-mortem Zahnstatus entspricht genau ante mortem Zahnstatus).*
- ☐ *... sonstige Methoden wegfallen und Zähne beständig sind (siehe postmortem Zustand des Leichnams).*
- ☐ *... es sich um eine günstige Methode handelt.*
- ☐ *... Sonstiges:*

☐ ***Nein, weil...***

- ☐ *... es sich um eine arbeitsaufwändige Methode handelt (Anfordern von antemortem Zahnstatus).*
- ☐ *... die Mithilfe von externen Institutionen notwendig ist (Zahnärzte / -innen).*
- ☐ *...Sonstiges:*

15. Zur eindeutigen Identifizierung eines unbekannten Toten ist ein Vergleich post-mortem und ante-mortem Status erforderlich. Wie wird in Ihrer Organisationseinheit der post-mortem Zahnstatus bei einem unbekannten Leichnam erhoben?

Mehrfachnennungen möglich

- ☐ *Eigene Polizeiinterne Kräfte*
- ☐ *Rechtsmedizin (Obduktion)*
- ☐ *Externe Spezialisten (z.B. spezialisierte Zahnärzte/-innen)*
- ☐ *Andere:*

16. Wie erfolgt die Kontaktaufnahme zu Zahnärzten/innen zwecks Abgleich post-mortem?

Mehrfachnennungen möglich

- ☐ *Printmedien (z.B. Zeitschriften wie die Zahnärztliche Mitteilungen)*
- ☐ *Digitale Medien*
- ☐ *Fachkreise (z.B. Arbeitskreis für forensische Odontostomatologie)*
- ☐ *Andere:*

17. Haben Sie einen festen Ansprechpartner für die zahnärztliche Identifikation?

Zum Beispiel einen Zahnarzt/ eine Zahnärztin

☐ *Ja* *-> Bei Auswahl „Ja" folgt Frage 18 für die Befragten*
☐ *Nein* *-> Bei Auswahl „Nein" folgt direkt Frage 19 für die Befragten*

18. Welche Ausbildung hat diese/r Zahnarzt/-ärztin?

☐ *Forensische/r Odonto-Stomatologe/-in*
☐ *Allgemeine/r Zahnarzt/-ärztin*
☐ *Fachzahnarzt/-ärztin für Oralchirurgie*
☐ *Fachzahnarzt/-ärztin für Kieferorthopädie*
☐ *Mund-Kiefer-Gesichtschirurg/-in*
☐ *Andere:*

19. Wie oft erhalten Sie eine Rückmeldung?

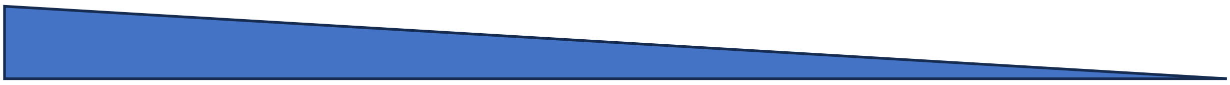

☐	☐	☐	☐	☐
Immer	*Oft*	*Manchmal*	*Selten*	*Nie*

20. Von welchen Faktoren ist eine Rückmeldung Ihrer Meinung nach abhängig?

Mehrfachnennungen möglich

☐ *Eindeutiger Zahnstatus post-mortem*
☐ *Gut geführte Patientenakten mit vollständigem Zahnstatus (ante-mortem)*
☐ *Moderne Praxen (Digitalisierung)*
☐ *Saisonabhängig (Sommer/Urlaubszeit)*
☐ *Einzelbefund*
☐ *Massenbefunde (siehe Katastrophen)*
☐ *Bessere Mundgesundheit der Bevölkerung und somit weniger zahnärztliche Behandlungen (weniger Zahnersatz, weniger Röntgenbilder)*
☐ *Andere:*

21. Welche Methode wird bei der zahnärztlichen Identifikation am häufigsten verwendet?
Mehrfachnennung möglich

- ☐ *Der Vergleich von postmortem – antemortem Zahnstaten m.H. des Dental Profiling (Untersuchungen der Zähne, Knochenstruktur, Mundhöhle, Weichteile, um ein Profil zu erstellen, Röntgenbilder, Zahnstatus)*
- ☐ *DNA-Gewinnung aus einem Zahn mit nachfolgender DNA-Analyse*
- ☐ *Altersdiagnostik*
- ☐ *Odontometrische Geschlechtsbestimmung*
- ☐ *Wissen über Medizinprodukte: z.B. Auslesen Chargennummer in Implantaten*
- ☐ *Andere:*

22. Wie hilfreich wäre eine digitale Plattform, um Zahnärzte/-innen zu erreichen?

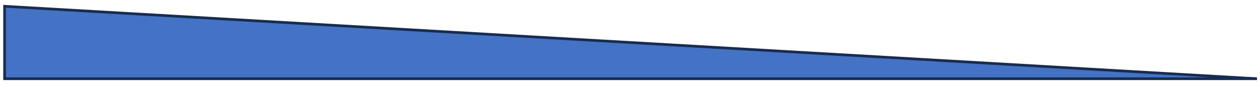

☐	☐	☐	☐	☐
Sehr hilfreich	*Etwas hilfreich*	*Neutral*	*Weniger hilfreich*	*Gar nicht hilfreich*

23. Was würde die primäre Anwendung der zahnärztlichen Identifikation beim Fund eines unbekannten Toten erleichtern?

- ☐ Register
- ☐ Datenblatt
- ☐ Zentraler Ansprechpartner pro Bundesland (Forensische/r Odontostomatologe/in)
- ☐ Sonstige:
