# Supplementary material for: Forensic dentistry for identity verification. A survey at the state police level
Source: Bundesgesundheitsblatt Gesundheitsforschung Gesundheitsschutz. 2023 Sep 27;66(11):1268–76. [Article in German] doi: 10.1007/s00103-023-03769-2 (PMC10622376; doi:10.1007/s00103-023-03769-2)
Supplement: Supplementary file 2 [file 103_2023_3769_MOESM2_ESM.pdf]

Onlinematerial 2

Tab. Z1: Anzahl der aufgefunden unbekannten Leichen im Zuständigkeitsbereich der Dienststelle

Leichenfunde	Anzahl der Angaben	Anteil (%)
> 5 pro Monat	6	9,7
bis zu 5 pro Monat	52	83,9
Keine Angabe	4	6,5
Gesamt	62	100,0

Tab. Z2: Inanspruchnahme kombinierter Identifizierungsmethoden

Kombination der Identifizierungsmethoden	Anzahl	Anteil (%)
Ja	45	72,6
Nein	13	21,0
Keine Angabe	4	6,5
Gesamt	62	100,0

*Tab. Z3: Nützlichkeit einer digitalen Plattform um Zahnärzt*innen zu erreichen*

Digitale Plattform	Anzahl	Anteil (%)
Gar nicht hilfreich	1	1,6
Weniger hilfreich	3	4,8
Neutral	6	9,7
Etwas Hilfreich	9	14,5
Sehr Hilfreich	35	56,5
Keine Angabe	8	12,9
Gesamt	62	100,0

Tab. Z4: Optimierungsansätze zur Erleichterung der Anwendung der forensischen Zahnmedizin

**Optimierungs-ansatz**	**Gewählt**	**Anteil (%)**	**Nicht gewählt**	**Anteil (%)**	**Gesamt**
Register	22	35,5	40	64,5	100,0
Datenblatt	8	12,9	54	87,1	100,0
Zentraler Ansprechpartner pro Bundesland (Forensische/r Odontostomatologe/in)	39	62,9	23	37,1	100,0
